# Non-coding genetic variation shaping mental health

**DOI:** 10.1016/j.copsyc.2018.07.006

**Published:** 2019-06

**Authors:** John P Quinn, Abigail L Savage, Vivien J Bubb

**Affiliations:** Department of Molecular and Clinical Pharmacology, Institute of Translational Medicine, The University of Liverpool, Liverpool L69 3BX, UK

## Abstract

Over 98% of our genome is non-coding and is now recognised to have a major role in orchestrating the tissue specific and stimulus inducible gene expression pattern which underpins our wellbeing and mental health. The non-coding genome responds functionally to our environment at all levels, encompassing the span from psychological to physiological challenge. The gene expression pattern, termed the transcriptome, ultimately gives us our neurochemistry. Therefore a major modulator of mental wellbeing is how our genes are regulated in response to life experiences. Superimposed on the aforementioned non-coding DNA framework is a vast body of genetic variation in the elements that control response to challenges. These differences, termed polymorphisms, allow for a differential response from a specific DNA element to the same challenge thus potentially allowing ‘individuality’ in the modulation of our transcriptome. This review will focus on a fundamental mechanism defining our psychological and psychiatric wellbeing, namely how genetic variation can be correlated with differential gene expression in response to specific challenges, thus resulting in altered neurochemistry which consequently may shape behaviour.

## Highlights

• Gene expression determined by the genome mediating a response to cell environment.
• Genetic variation results in distinct individual response in gene expression.
• Non-coding DNA is an important site for such functional genetic variation.
• Gene expression is a major modulator of brain chemistry and thus behavior.

**Current Opinion in Psychology** 2019, **27**:18–24

This review comes from a themed issue on **Genetics**

Edited by **Brian B Boutwell** and **Michael A White**

For a complete overview see the Issue and the Editorial

Available online 24th July 2018

**https://doi.org/10.1016/j.copsyc.2018.07.006**

2352-250X/© 2018 The Authors. Published by Elsevier Ltd. This is an open access article under the CC BY license (http://creativecommons.org/licenses/by/4.0/).

## Introduction

**Figure 1:**
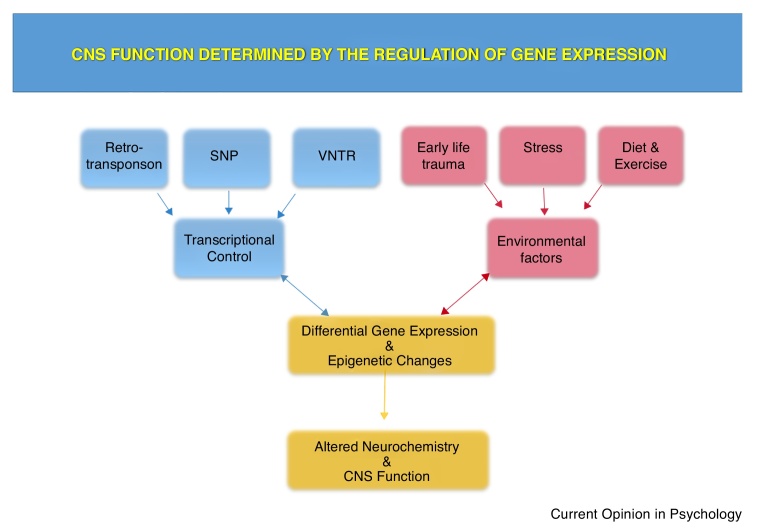
CNS function is determined by the regulation of gene expression.

The human genome has evolved to include a combination of both highly conserved regions of regulatory non-coding DNA (ncDNA) found across many species and human-specific regulatory DNA elements which together act to regulate expression of mRNA. This combination of DNA elements allows determination of where, when, how much and for how long, genes are expressed in the human brain in response to normal developmental, psychological and physiological cues, Figure 1. Many of these elements exhibit genetic variation which is not only associated with risk for a specific condition, but has also been demonstrated to alter the regulatory properties of the gene. The functional interpretation and analysis of ncDNA variation can be initially addressed *in silico* by overlaying its position on databases containing characterised and predicted functional elements within the genome, Box 1. The most easily accessible free database is the Encyclopaedia of DNA Elements (ENCODE; https://www.encodeproject.org/) which is a collaboration of research groups funded by the National Human Genome Research Institute [1,2^••^], this can be used in combination with a plethora of other database browsers [3] such as the University of California Santa Cruz (UCSC) Genome Browser (http://genome.ucsc.edu/) [4]. This review will begin with an introduction to the most conserved regulatory regions in the genome and how these may be functionally modified by the simplest and most extensively studied class of genetic variation, single nucleotide polymorphisms (SNPs). The review will then focus on human regulatory elements that are associated with neuropsychiatric conditions which are larger blocks of DNA variation such as variable number tandem repeats (VNTRs) and non-long terminal repeat (non-LTR) retrotransposons, Box 2.

Box 1 **Genome Browsers to interrogate DNA function.**

Encyclopaedia of DNA Elements (https://www.encodeproject.org/)

Evolutionary conserved regions browser (https://ecrbrowser.dcode.org/)

NIH Roadmap Epigenomics Consortium (http://www.roadmapepigenomics.org/)

University of California Santa Cruz (UCSC) Genome Browser (http://genome.ucsc.edu/)

Alt-text: Box 1

Box 2 **Genetic variation commonly found in the genome.**

**SNP: single nucleotide polymorphism**

The most common form of genetic variation in the genome. When located in a coding exon it may change the amino acid encoded thus altering the protein. In non-coding DNA it may have a variety of functions, however since transcription factors are sequence specific DNA binding proteins it could change the affinity or specificity of that interaction thus altering the function of a specific DNA element.

**VNTR: variable number tandem repeats**

These are adjacent repeats of single or more nucleotides and are a large component of the non-coding DNA. Often used in forensic DNA analysis as a genetic fingerprint given their variation in the genome. They are routinely separated into microsatellites (ranging in repeat length from 1 to 9 base pairs) and minisatellites (ranging in length from 10 to 60 base pairs), both classes are generally repeated 3-50 times. They are also found in exons such as in the Huntington gene.

**RTE: retrotransposon**

RTEs constitute approximately 42% of the human genome. Retrotransposons can be subdivided in long terminal repeat (LTR) retrotransposons (also termed ‘endogenous retroviruses’) and non-LTR retrotransposons lacking LTRs. Non-LTR retrotransposons propagate via a copy-and-paste mechanism, meaning that retrotransposon transcripts are reverse transcribed into a cDNA intermediate which is integrated into a new site of the host genome. Non-LTR retrotransposons can be sub-divided into LINE-1, *Alu* and SINE-VNTR-*Alu* (SVA) families which have the capacity to modify gene expression at transcriptional and post transcriptional levels. They constitute 21%, 13% and 0.2% respectively of the human genome.

Alt-text: Box 2

## Evolutionary conserved regions

Evolutionary conserved regions (ECRs) in the genome can be easily found using the ECR browser (https://ecrbrowser.dcode.org/) [5]. ECRs in this browser are typically defined as regions of sequence within the human genome that retain 70% or more sequence identity over a window of 100 bases when compared to the corresponding region of sequence in other species, this will frequently include exons in coding DNA. However, Pennacchio *et al.*, were amongst the first to demonstrate that ECRs in the non-coding DNA (ncECRs) could be important, particularly in directing gene expression in the CNS. They determined by use of a transgenic mouse model that whilst ncECRs could direct expression in a broad range of anatomical structures in the embryo, the majority of the ncECRs tested directed expression to various regions of the developing nervous system [6]. Subsequently, consistent with this, a third of paralogous ncECRs examined were predicted to have regulatory activity in the brain [7], for example, deletion of ncECRs in the neuronal transcription factor Arx resulted in substantial alterations of neuron populations and structural brain defects in a trangenic model [8]. Furthermore, the combinatorial complexity of gene expression was exquistely demonstrated in a transgenic model of craniofacial morphology in which the action of multiple ncECRs driving expression of many genes resulted in a vast array of facial differences [9^••^]. These studies demonstrated that ncECRs can have important transcripitonal regulatory properties, therefore the expectation is that polymorphism in such domains has the potential to modify interactions with transcription factors and thus affect regulatory function.

## Single nucleotide polymorphisms

Early studies of genetic variation correlated with mental health focused on DNA variation in exons encoding proteins. Most of these studies addressed SNPs; thus a SNP that changed an amino acid (non-synonymous change) or resulted in a truncation of the protein could be mechanistically relevant as it could alter protein function. However, with technological advances the ability to address SNPs in genome wide association studies (GWAS) rather than solely exons, demonstrated that the vast bulk of SNP variation associated with behavioural and psychiatric conditions was in ncDNA [10,11]. GWAS has led to significant discoveries in defining some of the genes involved in neuropsychiatric disorders and demonstrated there is genetic overlap between many of the major psychiatric disorders [12,13]. In several examples the proteins identified can work together to alter a key pathway underpinning wellbeing and mental health, such as those modifying calcium signalling [14].

**Figure 2:**
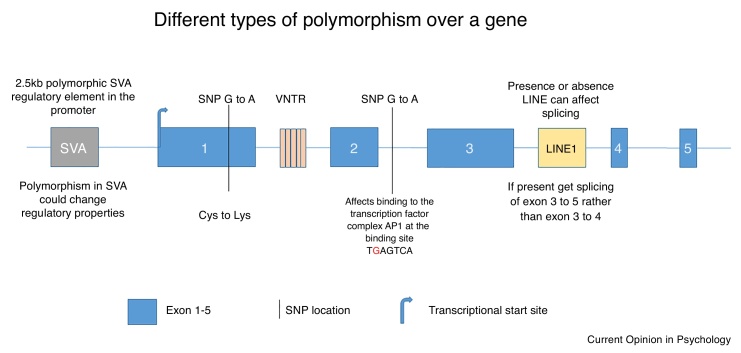
Different types of polymorphism over a gene.

Understanding the mechanistic significance of SNPs in ncDNA for a specific condition has been a much more difficult task than for SNPs found in exons. A SNP in ncDNA could be tagging a regulatory domain 10K+ bases from itself (a tagging SNP is representative of a large section of DNA that is inherited as one, thus the SNP is not the causative agent but rather highlights a region of DNA). Analysis of SNP variation within ENCODE and associated data sets can determine if it is present in a genomic region defined as a regulatory domain. In this scenario, the SNP could affect the efficiency or specificity with which a transcription factor, proteins which modulate the process of transcription, would bind to this regulatory DNA sequence, Figure 2.

We and others demonstrated that SNP polymorphisms in ncECRs which correlated with known behavioural problems could modify the regulatory properties of the ECR including those associated with depression located in BDNF, BICC1 and galanin genes [15, 16, 17, 18, 19]. Furthermore multiple ncECRs may be required for appropriate gene expression, for example eight conserved ncECRs were identified at the schizophrenia-associated MIR137/DPYD locus of these, six were shown to be positive transcriptional regulators, and two negative transcriptional regulators in a human cell line model [18,19]. Bioinformatic analysis of this locus using the Psychiatric Genomics Consortium GWAS dataset for schizophrenia highlighted five of the ncECRs had genome-wide significant SNPs in, or adjacent to their sequence [11].

Epigenetic marks which are indicative of active or inactive chromatin, are often found at regulatory DNA. Genetic variation such as GWAS risk SNPs, can effect such epigenetic parameters impacting on long term regulatory changes in response to challenge [20]. Both local (gene specific) and global (multigene) epigenetic changes have been implicated in neuropsychiatric disorders [21,22] and the NIH Roadmap Epigenomics Consortium (http://www.roadmapepigenomics.org/) data can be utilised to analyse such data. For example, local methylation variation at the glucocorticoid receptor gene has been associated with prenatal and postnatal depression [23^•^] and global differences in methylation in astrocytes have been associated with depression [24]. Simplistically, methylation of regulatory regions is considered a repressor of transcription as it interferes with transcription factor binding by limiting the accessibility of specific DNA recognition sequences. The ability to rapidly address genetic variation on a ‘road map’ of regulatory domains has allowed the development of a significantly better understanding of how the ncDNA GWAS SNPs can be mechanistically involved in mental health issues [25]. This can be further updated within the UCSC browser which permits new, novel data to be overlaid on the existing data from ENCODE.

## Variable number tandem repeats

SNP variation is not the only example of ncDNA variation that can affect the regulation of gene expression. Many of the best characterised genetic polymorphisms correlating with mental health issues are found in repetitive DNA, Figure 2. These include the VNTRs [26], examples of which have been identified in key behavioural and mental health-related genes. VNTRs have been demonstrated to be both biomarkers and transcriptional regulators in genes such as the serotonin transporter, the dopamine transporter and monoamine oxidase A [21,22,27, 28, 29, 30, 31, 32]. In these three examples, the primary DNA sequences of the VNTRs are rapidly evolving such that humans have their own specific VNTR sequences. All three of these monoaminergic genes contain a minimum of two VNTRs that have been demonstrated to act both independently and synergistically as transcriptional regulators whose function is further modulated by the repeat copy number within the VNTR [21,29,31]. The copy number of the repeat itself is also a biomarker for good mental health and wellbeing thus correlating function with phenotype [27,28,31,33,34]. Perhaps not unexpectedly VNTRs and GWAS SNPs in the same promoter may act additively or synergistically to regulate gene expression. This is exemplified by one of the promoters of the schizophrenia candidate risk gene, MIR137 [35,36^•^], where experimentally *in vitro*, the VNTR in the promoter can support differential reporter gene expression based on the copy number of the repeat within the VNTR, and inclusion of the promoter region encompassing the GWAS SNP can further modulate expression depending on the allele of the SNP present. This illustrates a route to identifying the potential functional significance of non-coding variants in transcriptional or post transcriptional regulatory mechanisms in areas distinct from the region of the DNA in which the GWAS SNP is found.

The rapid evolution of VNTRs has been noted more globally for contributing to primate evolution; analysis in humans and non-human great apes identified that genes with VNTRs have higher expression divergence than those without [37]. The association of VNTRs with gene expression is reflected in the finding that VNTRs are enriched in promoter regions and locations close to transcriptional start sites for mRNA expression [26,38,39]. Generally, VNTRs have not been analysed as extensively as SNPs which may be attributed to the requirement to perform PCR to genotype each VNTR target and the inability to accurately identify such regions in the initial short read whole genome sequencing protocols. Improved depth and coverage in whole genome sequence combined with the development of bioinformatic programmes such as ExpansionHunter may improve the association of VNTRs and other repeat variants with neuropsychiatric conditions [40].

## Non-LTR retrotransposons

Non-long terminal repeat (non-LTR) retrotransposons are mobile DNA elements that can copy and paste themselves into new genomic loci and are therefore polymorphic for their presence or absence at specific loci in the genome. These retrotransposable elements (RTEs), also known as ‘jumping genes’, can range in size from a few hundred to 6000 base pairs and have been shown to be major modulators of gene expression at several levels. Non-LTR retrotransposons comprise three classes, long interspersed nuclear element 1 (LINE-1), *Alu* and ‘SINE-VNTR-Alu’ (SVA), Figure 2. LINE-1 expression has been implicated in many neuropsychiatric conditions such as depression [41], addiction [42], schizophrenia [43, 44, 45] and autism [46]. The other two classes have also been implicated in CNS function, for example variation in the *Alu* sequence within an intron of the TOMM40 gene is associated with non-pathogenic cognitive decline [47] and X-Linked Dystonia-Parkinsonism is associated with the presence or absence of a SVA in the TAF1 gene [48^••^]. SVAs contain several distinct VNTR elements and have properties consistent with transcriptional regulation [49,50^•^,^51^].

There has been tremendous interest in RTEs, due to their ability to make a copy of themselves which then can reinsert at a different locus in the genome of that cell. Depending on when this occurs, it results in either novel heritable germline variation or somatic mutation that can alter cellular function in only the individual affected. The former generates a large reservoir of *de novo* genomic variation in the population which, to date, is poorly characterised as it is very seldom annotated properly in the DNA sequence databases. The mobilisation or ‘jumping’ of RTEs is proposed to increase both with age within the adult CNS and to comprise one of the key mechanisms underpinning age-related CNS problems [52^••^,^53^,54^•^]; several instances where this variation has been addressed, have associated it with disease [48^••^,^55^]. More recently bioinformatic analysis has improved to allow robust identification of RTEs using programmes such as TEBreak (https://github.com/adamewing/tebreak) and MELT [56^•^].

## Development

The basic transcriptional mechanisms outlined in this review will also operate during development. Conditions such as schizophrenia and autism are often referred to as having a neurodevelopmental origin [57,58]. Modulation of the transcriptome during development could have a significant effect on the wiring of the brain and therefore how information is processed in the future. Early *in vivo* work using a mouse transgenic model indicated how a human serotonin transporter VNTR could differentially affect gene expression in key areas of serotonergic lineage based on the copy number of the repeat unit [32,59]. The regulation of regulatory domains during development will be determined by the co-expression of transcription factors as exemplified by the schizophrenia associated gene CACNA1C whose expression in development mirrors that of the transcription factor EZH2 an important regulator of the CACNA1C gene promoter [14]. An argument can be made that alterations in the transcriptome at specific times in foetal development could result in a physical change in neuronal connections that would be more difficult to correct than transcriptome changes in the adult [60].

## Summary

The identification of variation in the non-coding part of the genome which affects the regulation of gene expression in part explains the often episodic nature of mental health conditions. In addition, it offers the potential for resolution of these conditions by a variety of interventions ranging from pharmaceutical to cognitive behavioural therapy which modify the signalling pathways targeting specific gene regulatory domains, modulation of the ‘stress’ driving such pathways would alter the transcriptome and hence brain chemistry, Figure 1. A prior exposure to trauma or stress could leave a molecular scar of that event, represented by an epigenetic change which alters parameters of transcriptional or post transcriptional regulation in the medium to long term [61]. It is often considered that the environmental challenge needed to affect mental health should be severe, which is not necessarily correct. For example ‘normal’ child development could also have an effect on mental health and wellbeing [23^•^,^27^,^33^,^62^]. Similarly a more general approach to maintaining good mental health via diet and exercise could play a role as they could affect the cellular signalling pathways that affect mental health [63,64]. However these issues only illustrate the complexity of defining ‘life style/environment’ and its effect on our wellbeing given the complex nature of life-long experiences in defining our transcriptome, which in turn affects the neurochemistry that ultimately shapes CNS function. It’s often said that the genome is the roadmap through which ‘life style’ shapes the individual, however one can argue that the roadmap is unique for each one of us and we all have our own route to travel [65].

## Conflict of interest

None of the authors have interests to declare, thus we are stating officially ‘Declarations of interest: none'.

## References and recommended reading

Papers of particular interest, published within the period of review, have been highlighted as:

• of special interest
•• of outstanding interest
